# Long-term weight changes are associated with initial weight changes after nonalcoholic fatty liver disease diagnosis

**DOI:** 10.1097/HC9.0000000000000044

**Published:** 2023-02-09

**Authors:** Jacob V. DiBattista, Anna S. Lok, Vincent L. Chen

**Affiliations:** 1 Department of Internal Medicine, University of Michigan, Ann Arbor, Michigan, USA; 2 Division of Gastroenterology and Hepatology, Department of Internal Medicine, University of Michigan, Ann Arbor, Michigan, USA

## Abstract

Weight loss is the mainstay of treatment of NAFLD, but longitudinal data on weight changes and their impact on liver disease are limited. We aimed to characterize weight trajectory up to 5 years of follow-up, effects of weight loss on liver enzyme levels, and predictors of weight loss in NAFLD.

**Methods:**

This was a single-center retrospective study of consecutive patients with hepatic steatosis diagnosed on imaging, liver biopsy, or transient elastography between 2010 and 2020. The primary outcome was ≥5% weight change at 1–2 years. Secondary outcomes were weight change at 4–5 years and alanine aminotransferase level at 1–2 and 4–5 years. We conducted multivariable logistic regression to identify predictors of ≥5% weight loss at 1–2 years.

**Results:**

We included 11,559 patients with NAFLD. At year 1–2, 27% had ≥5% weight loss, whereas 26% had ≥5% weight gain. Total 59% and 68% of patients with weight loss and gain, respectively, sustained their weight change by year 4–5. Patients with weight loss at year 1–2 had lower alanine aminotransferase levels at year 1–2 and 4–5. Predictors of ≥5% weight loss included female sex, severe obesity, diabetes, and consultation with a dietitian or pharmacist.

**Conclusions:**

Over half of patients with NAFLD had ≥5% weight loss or gain within 1–2 years, and these changes were usually sustained at 4–5 years. Intensive intervention early after NAFLD diagnosis may result in long-term weight loss and decreased NAFLD disease activity.

## Graphical abstract

**Figure:**
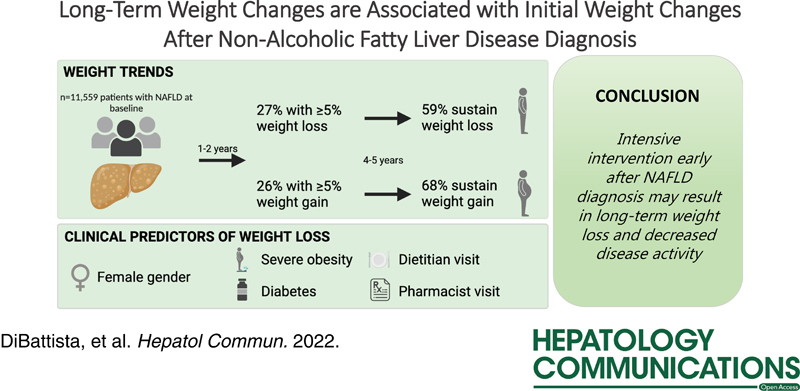

## STUDY HIGHLIGHTS

**WHAT IS KNOWN**

- Weight loss is key to treatment in NAFLD.
- In short-term (<2 y) follow-up, 20%–40% of patients with NAFLD attain ≥5% weight loss.

**WHAT IS NEW HERE**

- Among patients with short-term weight loss or gain, around 60% sustained the weight loss/gain at years 4–5.
- Weight loss was associated with decreased alanine aminotransferase levels.
- Predictors of weight loss include more severe obesity, diabetes, female sex, and dietitian visits.

## INTRODUCTION

NAFLD is the hepatic manifestation of metabolic syndrome and has become one of the leading causes of liver disease and cirrhosis worldwide.^1^,^2^ Currently, there are no US Food and Drug Administration–approved medications for NAFLD.^3^,^4^ The cornerstone of NAFLD therapy recommended by the American Association for the Study of Liver Diseases and the American Gastroenterological Association is weight loss through lifestyle modifications.^5^,^6^ A 3%–5% weight loss can decrease hepatic steatosis, and a 7%–10% weight loss can reduce NASH and fibrosis.^7^ One recent analysis of 421 clinical trial participants found that a dose-dependent decrease in alanine aminotransferase (ALT), resolution of NASH, and improvement in fibrosis with increased weight loss.^8^

Although guidelines recommend a combined approach with both diet and exercise to achieve target weight loss, the optimal strategies to promote weight loss or predict who will achieve weight loss are not known.^9^–^12^ Numerous studies have evaluated weight trends in patients with NAFLD. However, the majority of studies involve either placebo control groups in clinical trials with a high rate of placebo response or observational studies of cohorts recruited from a subspecialty hepatology clinic, which represent populations undergoing close monitoring and counseling.^13^–^15^ In addition, many published studies have follow-up periods of <1–2 years, so the sustainability of weight changes is less understood.

Our study sought to analyze weight trends in patients with NAFLD over a 5-year follow-up period using data from a cohort of patients with hepatic steatosis identified on imaging in a large health care system to determine the incidence, sustainability, and clinical predictors of weight loss.

## PATIENTS AND METHODS

### Ethical statement

This study was considered exempt by the Institutional Review Board of University of Michigan and a waiver of informed consent was obtained. All study procedures were conducted in accordance with the Declarations of Helsinki and Istanbul.

### Cohort design

**FIGURE 1:**
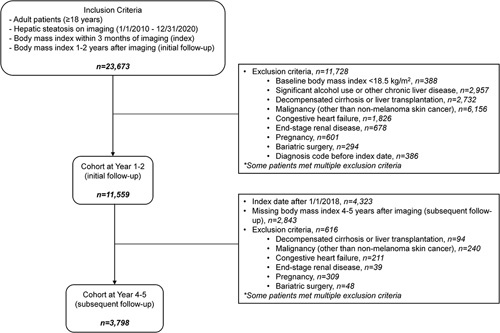
Cohort design flow diagram.

This was a retrospective cohort study that assessed weight changes in patients with NAFLD over a 5-year period. The cohort design is summarized in Figure 1. We screened patients seen at Michigan Medicine, a large, integrated academic health care system in southeastern Michigan, with abdominal imaging reports (ultrasound, computed tomography, or magnetic resonance imaging) between January 1, 2010, and December 31, 2020. Inclusion criteria were adults (age ≥18 y), hepatic steatosis on imaging, body mass index (BMI) data within 3 months of imaging, and at least 1 additional BMI measurement 1–2 years after imaging. An imaging report was classified as showing hepatic steatosis if it met the following criteria: (1) included the term “steato” or “fat” in the findings or impression section, (2) the term appeared in the same sentence as the term “hepat” or “liver,” and (3) the term did not appear in the same sentence as a negative term such as “no” or a term indicating evaluation such as “rule out” or “assess for.” Exclusion criteria were baseline BMI <18.5 kg/m^2^, significant alcohol use or other chronic liver disease, and conditions associated with large or rapid weight fluctuations, namely decompensated cirrhosis or liver transplantation, malignancy (other than nonmelanoma skin cancer), congestive heart failure, end-stage renal disease, pregnancy, or prior bariatric surgery (Supplemental Table 1, http://links.lww.com/HC9/A100). To ensure this was the first diagnosis of NAFLD, we excluded patients with an International Classification of Diseases (ICD) code for NAFLD >6 months before the index imaging date; the reason for the 6-month window is to not exclude patients who providers suspected NAFLD (even if it had not been confirmed) and entered an ICD code for NAFLD for that reason.

A subset of patients had an additional BMI measurement 4–5 years after imaging in addition to the 1–2 year data.

### Timeline and definitions

The first date when an imaging showed hepatic steatosis with an associated BMI within 3 months of the imaging was defined as the “index date.” The first period of BMI measurement at year 1–2 was defined as “initial follow-up.” The second period of BMI measurement at year 4–5, if available, was defined as “subsequent follow-up.” The “study period” was defined as the time between the index date and the last follow-up.

### Definition of outcomes

The primary outcome was weight change at initial follow-up (1–2 y) as compared with baseline, categorized as 3 groups: (1) weight loss (≥5% decrease), (2) stable weight (<5% change), and (3) weight gain (≥5% increase). Secondary outcomes were (1) weight change at subsequent follow-up (4–5 y) and (2) ALT at initial follow-up and subsequent follow-up, if available. ALT values were categorized as <1x, 1–2x, and>2x upper limit of normal (ULN), with cutoffs for females and males defined as 19 and 30 U/L, respectively. Finally, we evaluated predictors of weight loss at initial follow-up.

### Data processing

We collected all outpatient BMI values from 3 months before the index date to the end of the study period (year 5) or the last available clinical encounter. We excluded implausible BMI values (BMI <10 or >100 kg/m^2^). We defined baseline BMI as the mean BMI value around the index date (±3 mo), the BMI at initial follow-up as the BMI value closest to year 2 from the index date, and BMI at subsequent follow-up as the BMI value closest to year 5 from the index date. ALT values at each timepoint were defined in a similar manner.

Clinical information, including demographics, comorbidities, laboratory studies, and visits to selected specialist clinics were collected by automated electronic medical record extraction. Medical diagnoses were defined using ICD-9 or-10 codes (Supplemental Table 1, http://links.lww.com/HC9/A100).

### Statistical analyses

For descriptive statistics, mean and SE for continuous variables and frequency and proportion for categorical variables are presented. Continuous variables were compared with a Student *t* test if normally distributed and with Wilcoxon rank-sum test if not, and categorical variables were compared with a Chi-square test. For all comparisons, a 2-sided *p*-value of <0.05 was used to determine the statistical significance. Clinical predictors of weight loss at year 1–2 were determined through univariable logistic regression analysis, and all predictors with *p*<0.05 in univariable analysis were included in a multivariable model with the exception of medical subspecialties because they are not available in many practice settings. All analyses were performed using R version 4.0.2 (Vienna, Austria).

### Sensitivity analyses

We performed several sensitivity analyses. First, we included only patients with an ICD code for NAFLD (Supplemental Table 1, http://links.lww.com/HC9/A100). Second, we used mean BMI values around each follow-up period (rather than closest BMI values as in the primary analysis). Third, we required an additional BMI value around 1 year after the index date. Fourth, we limited the analysis to only patients who had both a year 4–5 BMI value and a year 1–2 BMI value. Fifth, we required a 7.5% or 10% weight change to count as weight change (vs. 5% change in the primary analysis). Finally, we excluded patients taking medications that may cause weight changes (Supplemental Table 2, http://links.lww.com/HC9/A100).^16^

## RESULTS

### Cohort

Our initial screen identified 23,673 patients that met inclusion criteria, of whom 11,559 patients were included in the cohort for assessment of weight changes at year 1–2 (initial follow-up). About half of the patients were excluded due to malignancies. Roughly one-third (3915) patients in the initial follow-up cohort had a BMI value at year 4–5 and were included in the subsequent follow-up cohort. Notably, about half of the patients who did not have a year 4–5 BMI value had an index date in 2018 or later, meaning they had not reached year 4 of follow-up at the time of data analysis (Figure 1).

Baseline characteristics of patients in the initial follow-up cohort are shown in Table 1. Mean age was 51.3 years; 51.0% were females; and 78.8% identified as White. There was a high prevalence of diabetes mellitus, dyslipidemia, and hypertension; and 63% patients had obesity. Mean aspartate aminotransferase, ALT, LDL, and hemoglobin A1c were all above normal. Patients with weight loss at year 1–2 were at baseline older and more often had diabetes, hypertension, and obesity class 2–3 than those with stable weight or weight gain (*p*<0.0001 for all).

**Table 1:** Patient characteristics at index date, stratified by weight category at initial follow-up

	Index	Initial follow-up (year 1–2)			
Characteristic	Total, n=11,559 (%)	Weight loss, n=3169 (%)	Stable weight, n=5380 (%)	Weight gain, n=3010 (%)	*p*
Demographics					
Age (y)	51.3 (15.5)	51.4 (15.4)	52.7 (14.8)	48.6 (16.5)	<0.001
Female sex	51.0	53.6	47.1	55.3	<0.001
Race					
Asian	5.3	4.5	6.5	3.9	<0.001
Black	7.9	7.9	7.6	8.3	<0.001
Hispanic	4.6	4.4	4.4	5.3	<0.001
White	78.8	80.1	77.8	79.2	<0.001
Other	3.4	3.1	3.7	3.3	<0.001
Comorbidities					
Diabetes mellitus	32.5	36.0	31.9	29.9	<0.001
Dyslipidemia	51.6	51.6	54.0	47.3	<0.001
Hypertension	58.0	60.1	58.2	55.2	<0.001
Coronary artery disease	17.6	18.4	17.2	17.3	<0.001
Cerebrovascular disease	9.0	10.2	8.0	9.6	<0.001
Peripheral arterial disease	9.9	10.1	9.6	10.3	<0.001
Compensated cirrhosis	2.5	2.6	2.5	2.4	<0.001
Diagnosis code for NAFLD	41.8	42.9	41.9	40.3	<0.001
Body mass index	33.0 (7.5)	34.0 (7.9)	33.0 (7.2)	31.8 (7.4)	<0.0001
Body mass index category					
Normal	10.8	8.3	9.1	16.4	<0.001
Overweight	25.8	23.1	26.3	27.8	<0.001
Obese class 1	29.6	29.8	30.7	27.4	<0.001
Obese class 2	18.1	20.0	18.6	15.3	<0.001
Obese class 3	15.7	18.8	15.3	13.1	<0.001
Laboratory studies					
Aspartate aminotransferase (U/L), n=9365	34.9 (27.6)	35.0 (25.5)	34.4 (23.4)	35.5 (35.5)	0.31
Alanine aminotransferase (U/L), n=9385	44.9 (41.9)	44.1 (38.1)	45.4 (36.7)	45.0 (52.8)	0.48
Platelet (K/µL), n=9067	254.0 (71.7)	254.5 (73.2)	249.5 (68.0)	261.1 (75.3)	<0.0001
Triglycerides (mg/dL), n=5264	194.7 (223.0)	197.0 (167.5)	193.4 (254.0)	194.8 (208.6)	0.89
HDL (mg/dL), n=5205	48.2 (14.1)	47.5 (13.9)	47.9 (13.6)	49.4 (15.1)	0.001
LDL (mg/dL), n=4971	102.1 (33.9)	100.6 (34.2)	103.3 (33.8)	101.2 (33.8)	0.037
Hemoglobin A1c (%), n=4539	6.3 (1.4)	6.4 (1.5)	6.3 (1.3)	6.2 (1.4)	0.0051
Clinic visits					
Dietitian	12.0	14.8	10.1	12.6	<0.0001
Pharmacist	5.7	7.2	4.9	5.4	<0.0001
Hepatology	7.8	8.3	8.4	6.1	<0.0001
Endocrinology	8.7	8.5	8.0	10.3	<0.0001
Weight loss program	0.6	0.9	0.4	0.8	<0.0001

*Note:* Data reported as mean (SD) for continuous variables or percent for categorical variables.

### Weight trends

**FIGURE 2:**
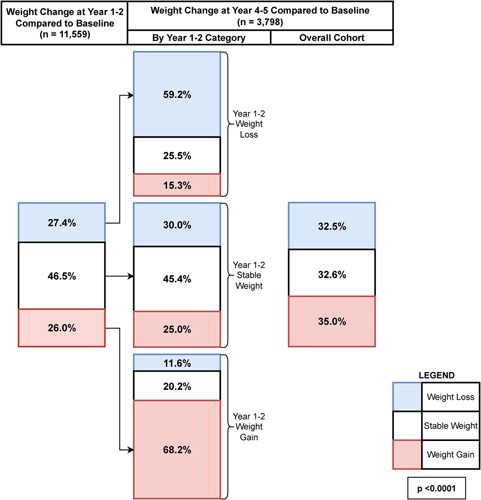
Weight trends at year 1–2 and year 4–5 follow-up compared with baseline in patients with NAFLD. Weight categories are defined as weight loss (≥5% weight decrease; blue), stable weight (<5% weight decrease or increase; white), and weight gain (≥5% weight increase; red). Values in the boxes show percentage of patients in each category. The leftmost column shows the overall distribution of weight category at year 1–2. The middle column shows the distribution of weight category at year 4–5, stratified by weight category at year 1–2. The rightmost column shows the overall distribution of weight category at year 4–5. *p*-value is by a chi-square test comparing distribution of weight category in year 4–5, stratified by weight category at year 1–2.

Weight trends for initial and subsequent follow-up periods are depicted in Figure 2. At year 1–2, 3053 patients (27.4%) had weight loss, 5589 patients (46.5%) had stable weight, and 3053 patients (26.0%) had weight gain. Patients with weight loss at year 1–2 were more likely (59%) to continue to sustain weight loss at year 4–5, whereas patients with weight gain were more likely (68%) to continue to sustain weight gain at year 4–5 (*p*<0.0001 for all). Among the patients with stable weight at year 1–2, slightly more lost than gained weight at year 4–5, 30.0% versus 25.0%. At year 4–5, roughly one third of patients fell into each of the three categories of weight changes (loss, stable, or gain) compared with baseline. Waterfall plots of percent change from baseline at years 1–2 and years 4–5 are shown in Supplemental Figure 1, http://links.lww.com/HC9/A100.

### Alanine aminotransferase trends

**FIGURE 3:**
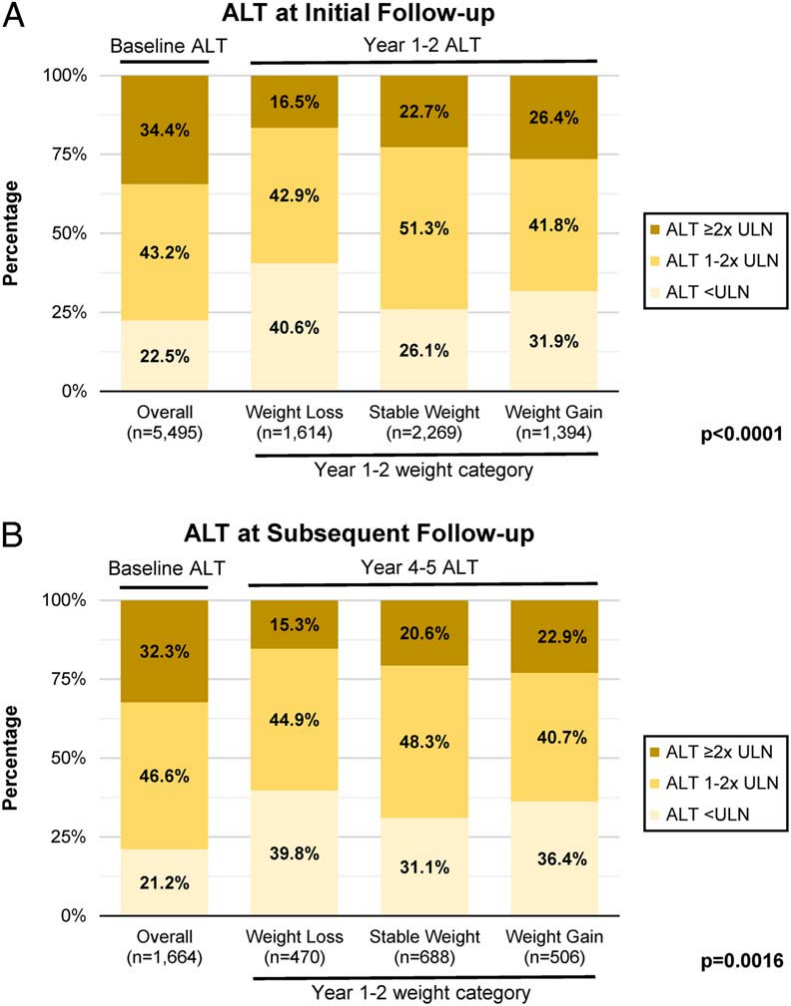
Alanine aminotransferase categories by weight change category at year 1–2 and year 4–5 follow-up in patients with NAFLD. (A) Alanine aminotransferase (ALT) levels at initial follow-up (1–2 y after index date). (B) ALT levels at subsequent follow-up (4–5 y after index date). From left to right, the 4 stacked bars depict the distribution of ALT categories among all patients at baseline (around the index date), patients with weight loss at year 1–2, patients with stable weight at year 1–2, and patients with weight gain at year 1–2. Note that only patients with an ALT level at year 1–2 or 4–5 were included in panels A or B, respectively. ALT categories defined as <ULN (upper limit of normal), 1–2x ULN, and >2x ULN with ULN cutoffs 19 and 30 U/L for women and men, respectively. *p*-values are for a chi-square test comparing distribution of ALT values at year 1–2 (for A) or year 4–5 (for B), stratified by weight loss category at year 1–2.

ALT trends for initial and subsequent follow-up periods are depicted in Figure 3. Patients with weight loss were more likely to have ALT <ULN at both follow-up periods, and patients with weight gain were more likely to have ALT≥2x ULN at both follow-up periods (*p*<0.005 for both). At both follow-up periods, the percentage of patients with ALT≥2x ULN increased in a stepwise manner from the group with weight loss, to the group with stable weight, and the group with weight gain. The inverse trend was not apparent with ALT<ULN. Regardless of weight changes, ALT values trended down during follow-up. Thus, the overall percentage of patients at year 1–2 and at year 4–5 with ALT<ULN was higher and the overall percentage with ALT>2x ULN was lower compared with baseline. At year 4–5, ALT distributions were similar in patients who had weight gain at year 1–2 that was sustained at year 4–5 and who initially had weight loss at year 1–2 but subsequently had weight increase to >5% above baseline by year 4–5 (Supplemental Figure 2, http://links.lww.com/HC9/A100).Similarly, those with weight loss at year 1–2 sustained to year 4–5 had similar ALT distributions to those with initial weight gain at year 1–2 but subsequent weight loss to >5% below their baseline by year 4–5.

### Sensitivity analyses

We conducted several sensitivity analyses. When we included only patients with known NAFLD, defined as having an ICD code for NAFLD, the results were similar to those of the primary analysis (Supplemental Figure 3, http://links.lww.com/HC9/A100). Using mean BMI values for each follow-up period to determine weight changes or requiring an additional BMI value around year 1 did not meaningfully affect the results (Supplemental Figure 4, http://links.lww.com/HC9/A100). We also included only patients with BMI values at both year 1–2 and 4–5 years, and the weight loss patterns at subsequent follow-up were similar to those of the entire cohort. Changing the definition of weight changes from 5.0% to 7.5% or 10% resulted in a smaller percentage of patients with weight changes. Using 10% to define weight changes, at year 1–2, weight loss occurred in 12%, stable weight in 77%, and weight gain in 11%; and at year 4–5, the corresponding percentages were 17%, 61%, and 17%, respectively (Supplemental Figure 5, http://links.lww.com/HC9/A100). Stability of the changes were similar with patients who lost weight at year 1–2 more likely to sustain or continue to lose weight at year 4–5, and patients who gained weight at year 1–2 more likely to sustain or continue to gain weight at year 4–5. Finally, excluding patients that took medications known to cause weight changes produced similar results (Supplemental Table 2, Supplemental Figure 6, http://links.lww.com/HC9/A100).

### Clinical predictors

The results from a logistic regression analysis looking at clinical predictors of weight loss at initial follow-up are detailed in Table 2. In the multivariable model, female sex, Medicare insurance, diabetes mellitus, obesity, and dietitian or pharmacist visit were predictive of year 1–2 weight loss (*p*<0.01 for all). As a sensitivity analysis, we evaluated the predictors of year 4–5 weight loss and found that the predictors were similar to the primary analysis, though in this sensitivity analysis age was and dietitian/pharmacist visits were not associated with weight loss in multivariable models (Supplemental Table 3, http://links.lww.com/HC9/A100). We also evaluated weight change, defined as percentage change from baseline weight, as a continuous variable rather than a categorical variable, and the predictors of weight change were similar to those in the primary analysis though diabetes and pharmacist visits were not significantly associated in multivariable models (Supplemental Table 4, http://links.lww.com/HC9/A100).

**Table 2:** Clinical predictors of weight loss at year 1–2 (initial follow-up)

	Univariable model		Multivariable model	
Predictor	OR (95% CI)	*p*	OR (95% CI)	*p*
Male sex (vs. female)	0.87 (0.80–0.94)	<0.0001	0.89 (0.82–0.96)	0.0052
Age category (y)				
<40	Referent	—	—	—
40–59	0.98 (0.89–1.09)	0.78	—	—
≥60	1.03 (0.92–1.14)	0.63	—	—
Race				
White	Referent	—	Referent	—
Asian	0.79 (0.65–0.96)	0.018	0.85 (0.70–1.03)	0.10
Black	0.98 (0.84–1.14)	0.81	0.88 (0.75–1.03)	0.10
Hispanic	0.91 (0.75–1.11)	0.37	0.88 (0.72–1.08)	0.21
Other	0.85 (0.67–1.07)	0.17	0.84 (0.67–1.07)	0.15
Insurance type				
Private	Referent	—	Referent	—
Medicaid	1.12 (0.96–1.30)	0.15	1.09 (0.94–1.28)	0.26
Medicare	1.23 (1.07–1.42)	0.0031	1.21 (1.05–1.39)	0.0080
Other	0.91 (0.83–1.00)	0.044	0.90 (0.82–0.99)	0.028
Comorbidities				
Diabetes mellitus	1.25 (1.14–1.36)	<0.0001	1.14 (1.04–1.25)	0.0067
Hypertension	1.13 (1.04–1.23)	0.0040	1.01 (0.92–1.10)	0.84
Dyslipidemia	1.00 (0.92–1.08)	0.97	—	—
Musculoskeletal diagnosis	1.12 (1.03–1.22)	0.0083	1.06 (0.97–1.16)	0.18
NAFLD ICD code	1.07 (0.98–1.16)	0.12	—	—
Weight category				
Normal	Referent	—	Referent	—
Overweight	1.21 (1.03–1.42)	0.018	1.23 (1.05–1.44)	0.013
Obese class 1	1.42 (1.22–1.66)	<0.0001	1.42 (1.21–1.66)	<0.0001
Obese class 2	1.62 (1.37–1.91)	<0.0001	1.56 (1.32–1.84)	<0.0001
Obese class 3	1.84 (1.55–2.17)	<0.0001	1.70 (1.43–2.02)	<0.0001
Clinic visits				
Dietitian	1.41 (1.25–1.59)	<0.0001	1.34 (1.18–1.51)	<0.0001
Pharmacist	1.44 (1.22–1.70)	<0.0001	1.33 (1.12–1.57)	0.0010
Hepatology	1.11 (0.96–1.29)	0.17	—	—
Endocrinology	0.96 (0.83–1.11)	0.61	—	—
Weight loss program	1.53 (0.95–2.45)	0.081	—	—

Clinic visits were defined as having a completed visit to the respective specialty before year 2 after the index date.

Abbreviations: ICD, International Classification of Diseases.

## DISCUSSION

In this study of nearly 12,000 patients with hepatic steatosis detected on imaging in a single health care system in the US, we found that only 27% had weight loss of ≥5% at 1–2 years though most patients sustained this weight loss at 4–5 years. Unfortunately, a similar proportion (26.0%) gained weight at 1–2 years and most sustained this weight gain at 4–5 years. Thus, at 4–5 years, roughly one third of patients were in each of the 3 categories of weight loss, stable weight, and weight gain. Notable variables predictive of weight loss were higher baseline BMI, diabetes, and having a clinic visit with a dietitian or pharmacist.

In contrast to other studies on this topic, we included consecutive patients with objective evidence of NAFLD, regardless of whether the patients or medical providers were aware of the diagnosis. Surprisingly, despite this difference, the 27% of patients in our cohort who attained ≥5% weight loss was largely concordant with other studies. A sensitivity analysis including only patients who carried a diagnosis of NAFLD showed a similar percentage (28%) achieving weight loss. The TARGET-NASH observational study of 2019 patients with NAFLD seen in gastroenterology/hepatology clinics in the US found that 32% of patients had 5% weight loss at any time during a median follow-up period of 39 months but did not report changes in ALT.^14^ In another US study of 924 patients with NAFLD followed in hepatology clinic, only 20% of patients lost 5% of their weight during short-term follow-up (median 11 mo) with no report of changes in liver enzymes.^17^ A recent meta-analysis of placebo arms of randomized-controlled trials of drugs aimed to reverse histological liver disease associated with NAFLD found that on average, BMI dropped only minimally (0.2 kg/m^2^) but AST and ALT dropped on average 6 and 10 U/L, respectively, after a follow-up period of 6–12 months for most studies.^15^ Thus, our findings imply that being followed in a gastroenterology or hepatology clinic, or even being enrolled in the placebo arm in a clinical trial that necessitates frequent visits, may have less impact on weight loss or liver enzyme changes than expected.

Our cohort was identified based on electronic medical record search for hepatic steatosis on imaging. Only 44% of patients had an ICD code for NAFLD, suggesting that many physicians who ordered the imaging tests may not have paid attention to the incidental finding of hepatic steatosis and many patients may not have been informed of the finding.^18^ Even among primary care providers and patients who knew of their diagnosis of NAFLD, understanding of NAFLD and the importance of lifestyle changes is limited.^19^,^20^ We did not find that having a diagnosis of NAFLD by ICD code was associated with a higher probability of weight loss in our cohort, but weight loss may be more common if a medical provider acts on that diagnosis, most notably by referring to dietitians or pharmacists. Other than these referrals, the predictors of NAFLD in our cohort included higher baseline BMI (which is expected as obesity is associated with a greater proportion of adipose tissue, which is easier to lose than lean mass), diabetes (likely because patients are more motivated to lose weight), and female sex, consistent with other studies.^14^

In this study, ALT levels tended to decrease over time in all weight change categories. We hypothesize that elevated liver enzymes due to NAFLD or abdominal complaints might have led to the initial imaging and the finding of hepatic steatosis while ALT levels during follow-up reflect steady-state levels. It should be noted that the biggest improvement in ALT levels (increase in percentage with normal ALT and decrease in percentage with ALT ≥2x ULN) was observed in those with weight loss, suggesting that weight loss has a beneficial effect on NAFLD activity.^7^

The most important finding from this study is that early weight trajectories appeared to be durable. In our study, among patients with initial weight loss, 59% still had ≥5% weight loss from baseline at subsequent follow-up (4–5 y after index date). The TARGET-NASH study’s results were similar in that only 21% of patients with initial weight loss had return of weight to baseline after a median of 32 months.^14^ The “optimistic” interpretation of these findings is that 60%–80% of patients with NAFLD who attained ≥5% weight loss sustained the weight loss for at least 3 years afterward. These findings on durability of weight loss are largely concordant with previous findings in other obesity and diabetes studies.^21^–^23^ Conversely, we found that 68% of patients who gained≥5% weight by years 1–2 sustained this weight gain by years 4–5, and only 12% had a year 4–5 weight ≥5% lower than that of their baseline weight.

On the basis of these findings, we advocate for intense interventions after the initial diagnosis of NAFLD. A new diagnosis may have a stronger motivating effect and a higher likelihood of success with counseling on lifestyle changes aimed at weight loss. We agree with previous studies that regular follow-up with primary care providers, dietitians, and other specialists is critical.^17^ Unfortunately, only 12% of patients in our cohort saw a dietitian within 1–2 years after the NAFLD diagnosis, even though seeing a dietitian was strongly associated with attaining ≥5% weight loss, which reflects a significant missed opportunity. Multidisciplinary NAFLD or obesity clinics may prove more effective in achieving sustained weight loss through a holistic personalized approach that includes nutritional counseling, fitness coaching, pharmacist-led management of chronic conditions such as diabetes, as well as use of weight loss medications and bariatric surgery or endoscopic procedures.^24^–^26^ Formal exercise and fitness training are effective in decreasing weight and ALT levels in patients with metabolic comorbidities but are rarely offered in NAFLD clinics.^27^ With the proliferation of online fitness programs, NAFLD clinics should consider adding fitness training to the armamentarium of NAFLD therapies.

This study has limitations in that patients were from a single tertiary care center and most were Caucasian. In addition, due to the retrospective nature of the study, we were not able to determine the indication for the imaging study that led to the detection of hepatic steatosis, whether patients were informed of the finding of hepatic steatosis, and, if they were, whether they were counseled on lifestyle changes. We were also unable to determine whether patients received nutritional counseling outside our health care system or participated in fitness programs after the finding of hepatic steatosis. Nevertheless, this study has several strengths including a relatively large cohort of consecutive patients (~12,000) with objective evidence of hepatic steatosis. Follow-up was longer than most existing studies in this field, including nearly 4,000 patients followed for 5 years. We were careful to exclude patients with medical conditions that cause weight fluctuations or who may have had a previous diagnosis of NAFLD, and we conducted a number of sensitivity analyses with similar findings, suggesting robustness of our results. Finally, we included all patients with NAFLD, not only those seen in hepatology clinic or enrolled in an intervention program, thus more closely resembling a “real-world” cohort.

Many patients with evidence of NAFLD remain unaware of their diagnosis and thus may miss the opportunity to receive counseling on appropriate lifestyle changes. Simple electronic medical record algorithms alerting physicians when imaging studies show steatosis may allow patients with NAFLD to undergo risk stratification and, if appropriate, undergo interventions aimed at early weight loss.^28^ Our findings suggest that earlier interventions aimed at early weight loss may result in improved long-term outcomes in patients with NAFLD: over half of patients with short-term weight loss sustained this weight loss at year 4–5. Early referrals to dietitians and, if available, multidisciplinary clinics may lead to durable, clinically impactful changes in weight and long-term outcomes.

## Supplementary Material
