# Supplementary material for: Long-term weight changes are associated with initial weight changes after nonalcoholic fatty liver disease diagnosis
Source: Hepatol Commun. 2023 Feb 9;7(3):e0044. doi: 10.1097/HC9.0000000000000044 (PMC9915952; doi:10.1097/HC9.0000000000000044)
Supplement: Supplementary file 1 [file hc9-7-e0044-s001.docx]

**Supplemental Table 1. Diagnosis codes and other definitions to identify study cohort and comorbidities**

| ***Variable*** | ***ICD-9 code(s)*** | ***ICD-10 code(s)*** |
| --- | --- | --- |
| *Exclusion criteria* |  |  |
| Other chronic liver disease or significant alcohol use | 291, 303, 305.0, 357.5, 425.5, 571.0, 571.1, 571.2, 571.3, 577.1, 790.3, 980, E860, 070.2, 070.3, 070.4, 070.5, 070.6, 070.7, 070.9, 273.4, 275.01, 275.1, 277.3, 571.42, 571.6, 576.1 | F10, G31.2, G62.1, G72.1, I42.6, K29.2, K70, K85.2, K86.0, R78.0, T51, Y90, Z50.2, Z72.1, B16, B17.0, B17.1, B17.9, B18, B19, B94.2, E83.0, E83.1, E85, E88.0, K74.3, K75.4, K83.0 |
| Liver transplantation or decompensated cirrhosis (cirrhosis plus any of the following: ascites, hepatic encephalopathy, or variceal bleeding) | V42.7, 996.82 | G94.3, I85, I86.4, I98.2, I98.3, K76.7, R18, R27.8 |
| Malignancy (other than non-melanoma skin cancer) | 140-209, except 173 | C00-C97, except C44 |
| Congestive heart failure or end-stage renal disease | 402, 416, 425, 428, 585.4, 585.5, 585.6, V42.0, V42.1, V45.1 | I11.0, I25.5, I27.0, I27.2, I42, I43, I50, I51.7, N18.4, N18.5, Y84.1, Z49, Z94.0, Z94.1, Z94.3, Z99.2 |
| Bariatric surgery | V45.86 | Z98.84 |
| Pregnancy | Positive urine or serum human chorionic gonadotropin |  |
| *Comorbidities* |  |  |
| Diabetes mellitus | 249, 250 | E10, E11, E12, E13, E14 |
| Dyslipidemia | 272 | E78 |
| Hypertension | 401, 402, 403, 404, 405, 437.2 | I10, I11, I12, I13, I14, I15, I67.4 |
| Compensated cirrhosis | 289.4, 571.2, 571.5, 571.6, 572.3 | K70.3, K71.7, K72.1, K74.3, K74.4, K74.5, K74.6, K76.6, R16 |
| Musculoskeletal disorders | 712, 714, 715.15, 715.16, 715.25, 715.26, 715.35, 715.36, 715.95, 715.96, 721, 722, 723, 724 | M05, M06, M10, M11, M16, M17, M43.0, M43.1, M47, M48.0, M50, M51, M54 |
| NAFLD ICD code | 571.8 | K75.81, K76.0 |

Abbreviations: ICD, International Classification of Diseases; NAFLD, nonalcoholic fatty liver disease.

**Supplemental Table 2. Weight change medications used in sensitivity analysis**

| ***Medication class*** | ***List of medications*** |
| --- | --- |
| Glucagon-like peptide-1 receptor agonists | Exenatide, albiglutide, dulaglutide, liraglutide, semaglutide |
| Sodium-glucose cotransporter-2 inhibitors | Empagliflozin, canagliflozin, dapagliflozin |
| Thiazolidinediones | Pioglitazone, rosiglitazone, metformin/pioglitazone, metformin/rosiglitazone, troglitazone, glimepiride/pioglitazone, glimepiride/rosiglitazone, alogliptin/pioglitazone |
| Sulfonylureas | Chlorpropamide, glimepiride, glipizide, glipizide/metformin, glyburide, glyburide/metformin, tolazamide |
| Meglitinides | Repaglinide, nateglinide |
| Insulin | Insulin analog-glargine, insulin analog-lispro, insulin analog-detemir, insulin-human isophane, insulin regular human, insulin NPH-Reg human insulin, insulin-human regular, insulin analog-aspart mixed, insulin analog-degludec, insulin analog-glulisine, insulin analog-lispro mixed, insulin-human NPH, insulin aspart (niacinamide), insulin analog-glargine/lixisenatide, insulin analog-degludec/liraglutide, insulin lispro in sodium chloride, insulin regular in sodium chloride, insulin-pork |
| Corticosteroids | Betamethasone, dexamethasone, methylprednisolone, prednisone, prednisolone |
| Selective serotonin reuptake inhibitors | Paroxetine |
| Atypical antidepressants | Mirtazapine |
| Tricyclic antidepressants | Amitriptyline, amitriptyline/chlordiazepoxide, amitriptyline/perphenazine, nortriptyline |
| First generation (typical) antipsychotics | Perphenazine, clozapine |
| Second generation (atypical) antipsychotics | Olanzapine, quetiapine, risperidone |
| Antiepileptic drugs | Carbamazepine, valproate, gabapentin, pregabalin |
| Beta blockers | Propranolol, hydrochlorothiazide/propranolol, atenolol, atenolol/chlorthalidone, metoprolol, hydrochlorothiazide/metoprolol |
| Other weight loss medications | Orlistat, phenteramine/topiramate, bupropion/naltrexone, lorcaserin |

List derived from https:/academic.oup.com/jcem/article/100/2/342/2813109

**Supplemental Table 3. Clinical predictors of weight loss at year 4-5 (subsequent follow-up)**

| **Predictor** | **Univariable model** | | **Multivariable model** | | |
| --- | --- | --- | --- | --- | --- |
|  | **Odds ratio**  **(95% CI)** | ***p*-value** | **Odds ratio**  **(95% CI)** | ***p*-value** |  |
| Male sex (vs. female) | 0.86 (0.76-0.98) | 0.022 | 0.87 (0.76-0.99) | 0.040 |  |
| *Age category* |  |  |  |  |  |
| <40 years | Referent |  |  |  |  |
| 40-59 years | 1.19 (1.01-1.41) | 0.037 | 1.08 (0.91-1.30) | 0.37 |  |
| ≥60 years | 1.53 (1.27-1.83) | <0.0001 | 1.39 (1.13-1.71) | 0.0017 |  |
| *Race* |  |  |  |  |  |
| White | Referent |  | Referent |  |  |
| Asian | 0.84 (0.62-1.15) | 0.29 | 0.95 (0.69-1.30) | 0.73 |  |
| Black | 1.00 (0.79-1.25) | 0.97 | 0.84 (0.66-1.07) | 0.15 |  |
| Hispanic | 0.95 (0.69-1.29) | 0.73 | 0.95 (0.69-1.31) | 0.75 |  |
| Other | 0.54 (0.35-0.83) | 0.0053 | 0.58 (0.37-0.90) | 0.016 |  |
| *Insurance type* |  |  |  |  |  |
| Private | Referent |  | Referent |  |  |
| Medicaid | 0.91 (0.70-1.18) | 0.48 | 0.95 (0.72-1.25) | 0.71 |  |
| Medicare | 1.51 (1.19-1.90) | 0.00058 | 1.31 (1.03-1.67) | 0.027 |  |
| Other | 1.08 (0.94-1.24) | 0.30 | 1.10 (0.95-1.26) | 0.21 |  |
| *Comorbidities* |  |  |  |  |  |
| Diabetes mellitus | 1.86 (1.63-2.12) | <0.0001 | 1.52 (1.32-1.76) | <0.0001 |  |
| Hypertension | 1.48 (1.29-1.69) | <0.0001 | 1.09 (0.93-1.27) | 0.30 |  |
| Dyslipidemia | 1.13 (0.99-1.29) | 0.067 | - | - |  |
| Musculoskeletal diagnosis | 1.15 (0.99-1.33) | 0.062 | - | - |  |
| NAFLD ICD code | 1.05 (0.93-1.20) | 0.43 | - | - |  |
| *Weight category* |  |  |  |  |  |
| Normal | Referent |  | Referent |  |  |
| Overweight | 1.32 (1.01-1.73) | 0.040 | 1.27 (0.97-1.67) | 0.080 |  |
| Obese class 1 | 1.85 (1.43-2.40) | <0.0001 | 1.71 (1.31-2.22) | <0.0001 |  |
| Obese class 2 | 2.24 (1.71-2.95) | <0.0001 | 2.06 (1.56-2.73) | <0.0001 |  |
| Obese class 3 | 2.87 (2.18-3.78) | <0.0001 | 2.47 (1.86-3.30) | <0.0001 |  |
| *Clinic visits* |  |  |  |  |  |
| Dietitian | 1.12 (0.92-1.35) | 0.25 | - | - |  |
| Pharmacist | 1.50 (1.16-1.95) | 0.0020 | 1.25 (0.96-1.64) | 0.097 |  |
| Hepatology | 1.34 (1.04-1.72) | 0.022 | - | - |  |
| Endocrinology | 1.18 (0.96-1.44) | 0.11 | - | - |  |
| Weight loss program | 1.93 (0.95-3.92) | 0.068 | - | - |  |

Clinic visits were defined as having a completed visit to the respective specialty prior to year 2 after the index date. CI, confidence interval; NAFLD, nonalcoholic fatty liver disease; ICD, International Classification of Diseases.

**Supplemental Table 4. Clinical predictors of percent weight change at year 1-2 (initial follow-up)**

| **Predictor** | **Univariable model** | | **Multivariable model** | | |
| --- | --- | --- | --- | --- | --- |
|  | **Effect**  **(95% CI)** | ***p*-value** | **Effect**  **(95% CI)** | ***p*-value** |  |
| Male sex (vs. female) | -0.14% (-0.44% to 0.17%) | 0.39 | - | - |  |
| *Age category* |  |  |  |  |  |
| <40 years | Referent |  | Referent |  |  |
| 40-59 years | -1.32% (-1.71% to -0.92%) | <0.0001 | -1.30% (-1.71% to -0.89%) | <0.0001 |  |
| ≥60 years | -1.77% (-2.19% to -1.34%) | <0.0001 | -2.01% (-2.48% to -1.53%) | <0.0001 |  |
| *Race* |  |  |  |  |  |
| White | Referent |  | Referent |  |  |
| Asian | -0.18% (-0.87% to 0.51%) | 0.61 | -0.71% (-1.41% to -0.02%) | 0.044 |  |
| Black | 0.24% (-0.34% to 0.82%) | 0.41 | 0.35% (-0.22% to 0.93%) | 0.23 |  |
| Hispanic | 1.22% (0.48% to 1.96%) | 0.0013 | 0.97% (0.23% to 1.71%) | 0.010 |  |
| Other | -0.03% (-0.89% to 0.82%) | 0.94 | -0.28% (-1.12% to 0.56%) | 0.52 |  |
| *Insurance type* |  |  |  |  |  |
| Private | Referent |  | Referent |  |  |
| Medicaid | 0.84% (0.25% to 1.43%) | 0.0053 | 0.45% (-0.14% to 1.05%) | 0.13 |  |
| Medicare | -0.83% (-1.38% to -0.29%) | 0.0026 | -0.50% (-1.05% to 0.04%) | 0.070 |  |
| Other | -0.00% (-0.35% to 0.35%) | 0.99 | -0.03% (-0.38% to 0.31%) | 0.85 |  |
| *Comorbidities* |  |  |  |  |  |
| Diabetes mellitus | -1.00% (-1.33% to -0.67%) | <0.0001 | -0.35% (-0.71% to 0.00%) | 0.051 |  |
| Hypertension | -0.67% (-0.98% to -0.35%) | <0.0001 | 0.38% (0.03% to 0.73%) | 0.036 |  |
| Dyslipidemia | -0.53% (-0.84% to -0.23%) | 0.00070 | 0.13% (-0.20% to 0.47%) | 0.44 |  |
| Musculoskeletal diagnosis | -0.23% (-0.55% to 0.09%) | 0.15 | - | - |  |
| NAFLD ICD code | -0.08% (-0.40% to 0.23%) | 0.60 | - | - |  |
| *Weight category* |  |  |  |  |  |
| Normal | Referent |  | Referent |  |  |
| Overweight | -2.33% (-2.89% to -1.78%) | <0.0001 | -2.18% (-2.74% to -1.63%) | <0.0001 |  |
| Obese class 1 | -3.25% (-3.80% to -2.71%) | <0.0001 | -3.15% (-3.70% to -2.60%) | <0.0001 |  |
| Obese class 2 | -3.69% (-4.27% to -3.10%) | <0.0001 | -3.70% (-4.29% to -3.10%) | <0.0001 |  |
| Obese class 3 | -4.59% (-5.20% to -3.98%) | <0.0001 | -4.73% (-5.36% to -4.11%) | <0.0001 |  |
| *Clinic visits* |  |  |  |  |  |
| Dietitian | -1.04% (-1.52% to -0.57%) | <0.0001 | -0.95% (-1.42% to -0.48%) | <0.0001 |  |
| Pharmacist | -0.59% (-1.26% to 0.08%) | 0.086 | - | - |  |
| Hepatology | -0.95% (-1.53% to -0.38%) | 0.0012 | - | - |  |
| Endocrinology | 0.40% (-0.14% to 0.95%) | 0.15 | - | - |  |
| Weight loss program | -0.98% (-2.92% to 0.95%) | 0.32 | - | - |  |

Clinic visits were defined as having a completed visit to the respective specialty prior to year 2 after the index date. CI, confidence interval; NAFLD, nonalcoholic fatty liver disease; ICD, International Classification of Diseases.

**Supplemental Figure 1. Waterfall plots depicting percent change in weight from baseline at year 1-2 (A) and at year 4-5 (B).**


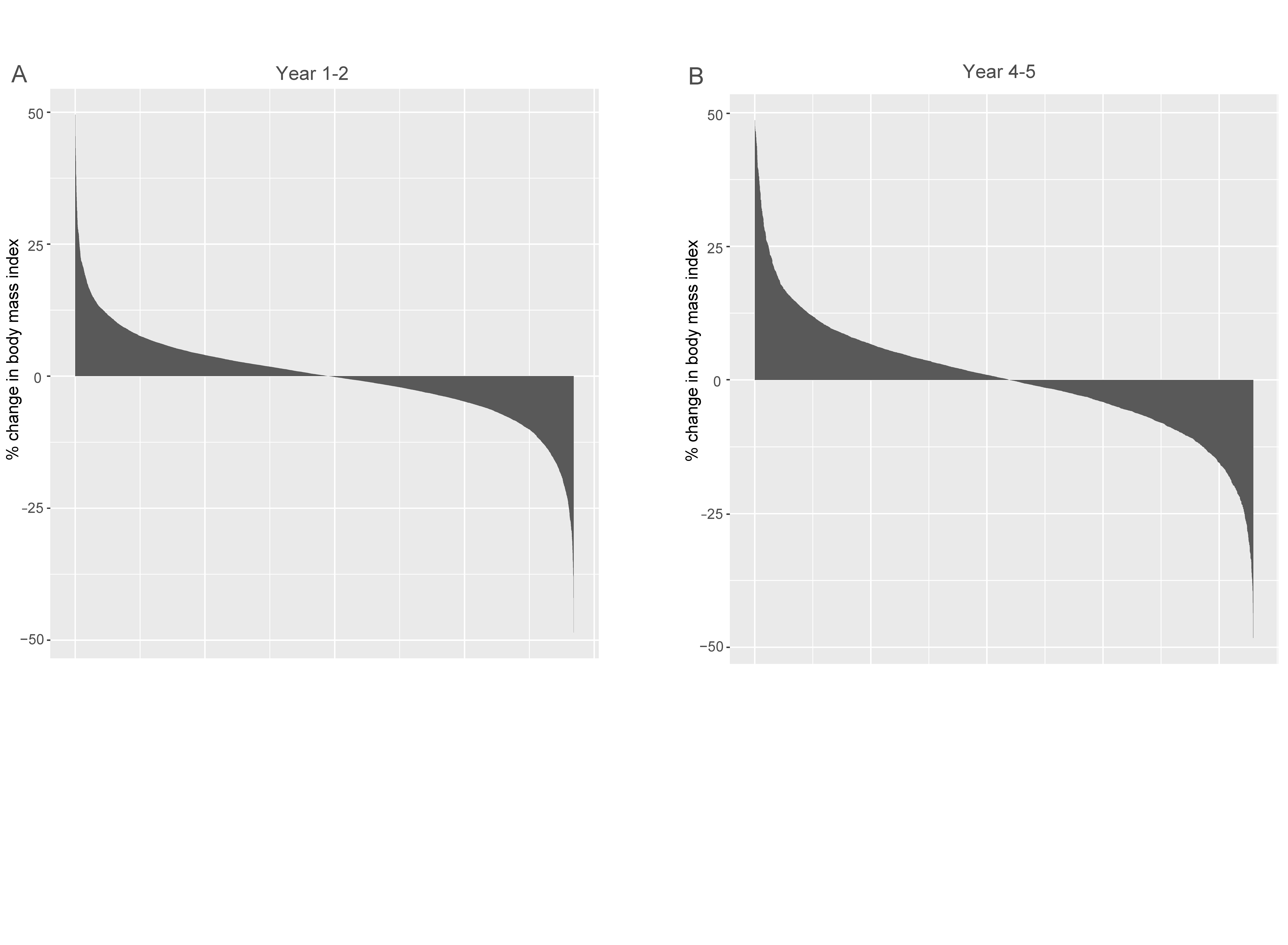


**Supplemental Figure 2. Alanine aminotransferase categories by weight change category at year 4-5 in patients with nonalcoholic fatty liver disease.**
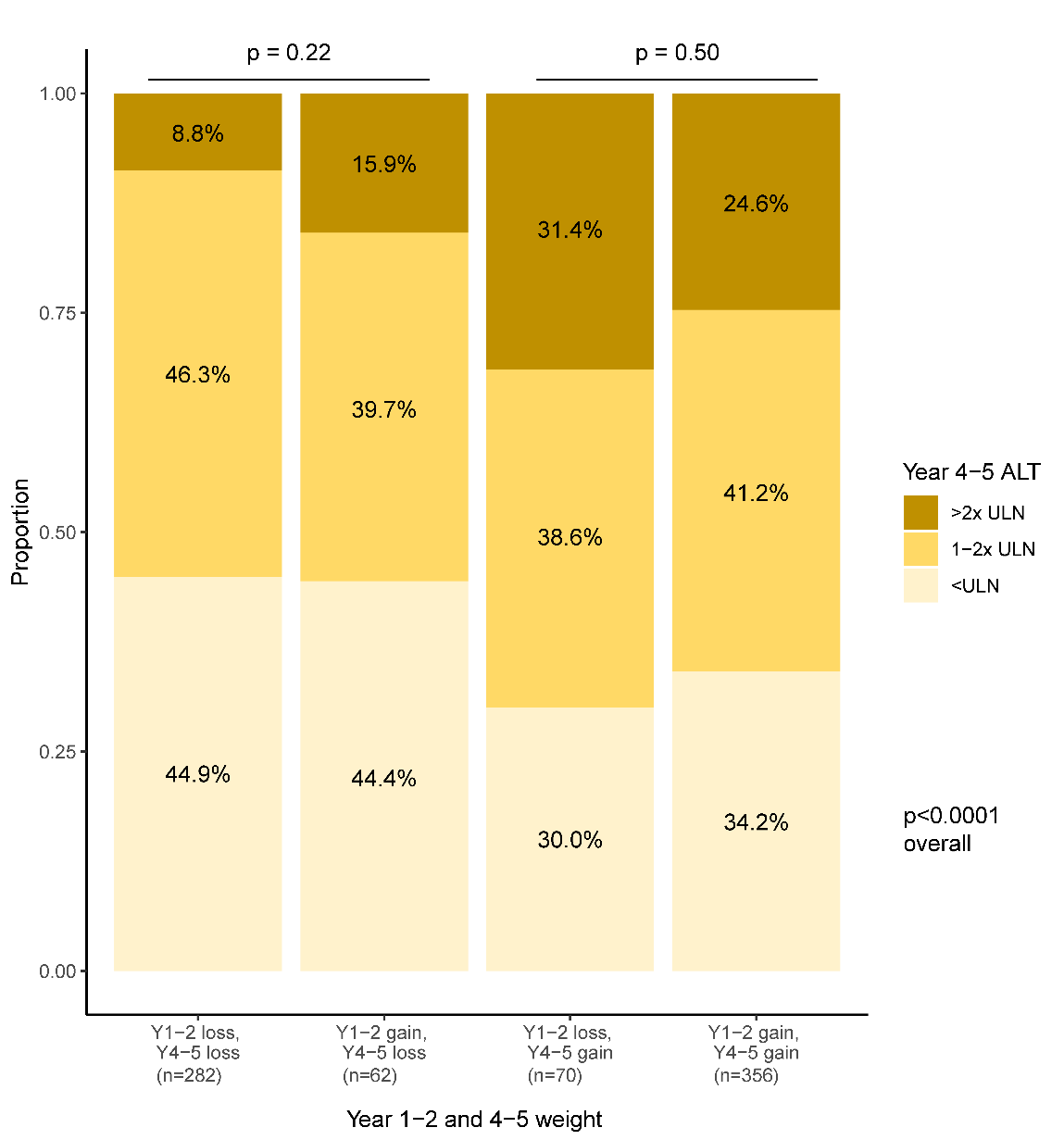


From left to right, the four stacked bars depict the distribution of ALT categories among patients with weight loss at year (Y) 1-2 and sustained weight loss at Y4-5, patients with weight gain at Y1-2 with subsequent change to >5% below baseline weight, patients with weight loss at Y1-2 with subsequent change to >5% above baseline weight, and patients with weight gain at Y1-2 and subsequent weight gain at Y4-5. ALT categories defined as <ULN (upper limit of normal), 1-2x ULN, and >2x ULN with ULN cutoffs 19 and 30 U/L for women and men, respectively. P values at the top of the figure are for pairwise chi-square tests, and the p value at the bottom right of the figure is for the overall distribution.

**Supplemental Figure 3. Weight trends at year 1-2 and year 4-5 follow-up compared to baseline in patients with nonalcoholic fatty liver disease - sensitivity analysis including only patients with a diagnosis code for nonalcoholic fatty liver disease**


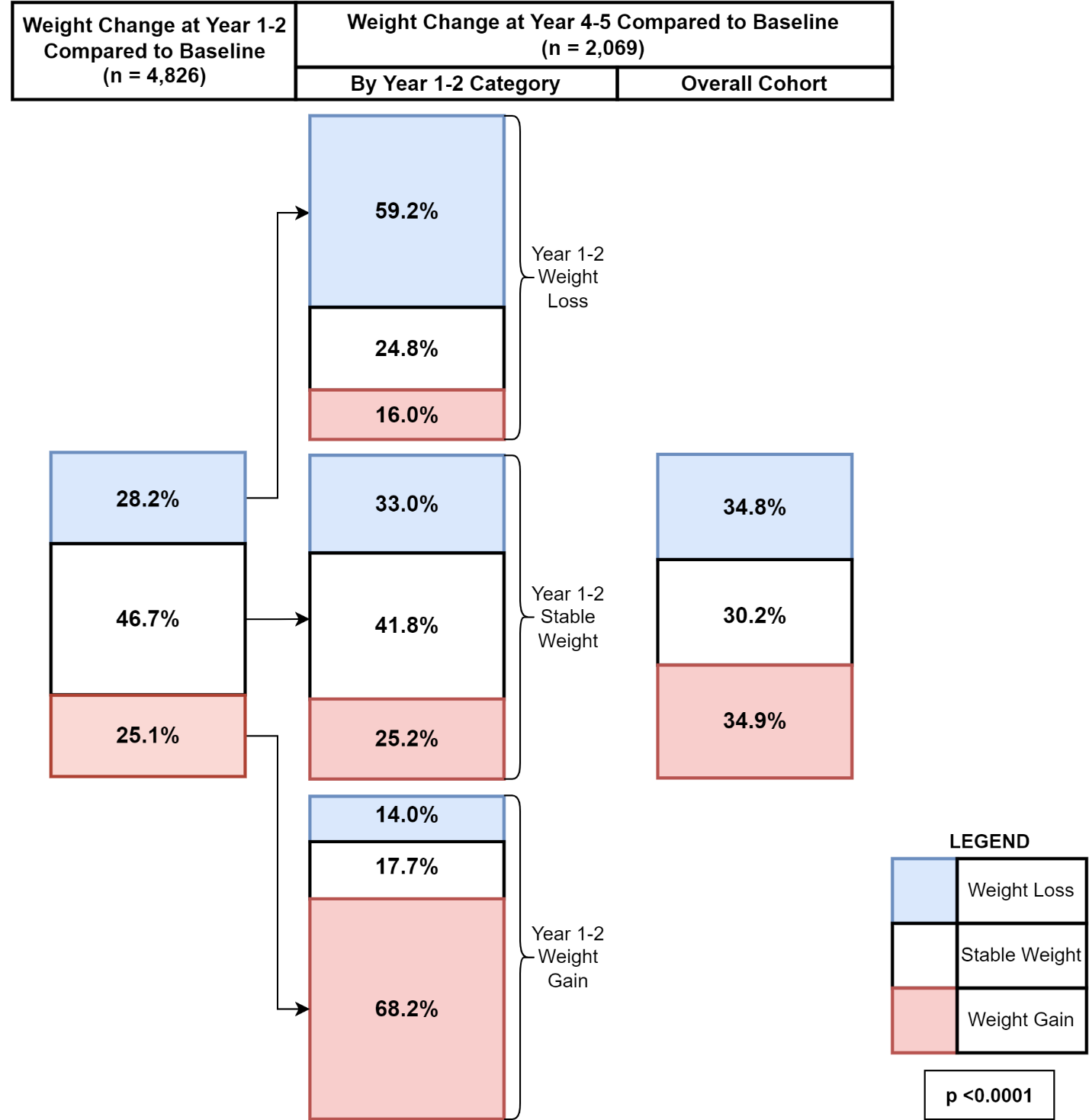


Weight categories are defined as weight loss (≥5% weight decrease; blue), stable weight (<5% weight decrease or increase; white), and weight gain (≥5% weight increase; red). Values in the boxes show percentage of patients in each category. The leftmost column shows the overall distribution of weight category at year 1-2. The middle column shows the distribution of weight category at year 4-5, stratified by weight category at year 1-2. The rightmost column shows the overall distribution of weight category at year 4-5. P value is by a chi-square test comparing distribution of weight category in year 4-5, stratified by weight category at year 1-2.

**Supplemental Figure 4. Weight trends at year 1-2 and year 4-5 follow-up compared to baseline in patients with nonalcoholic fatty liver disease - sensitivity analysis using mean values of all weight measurements during the follow-up period**


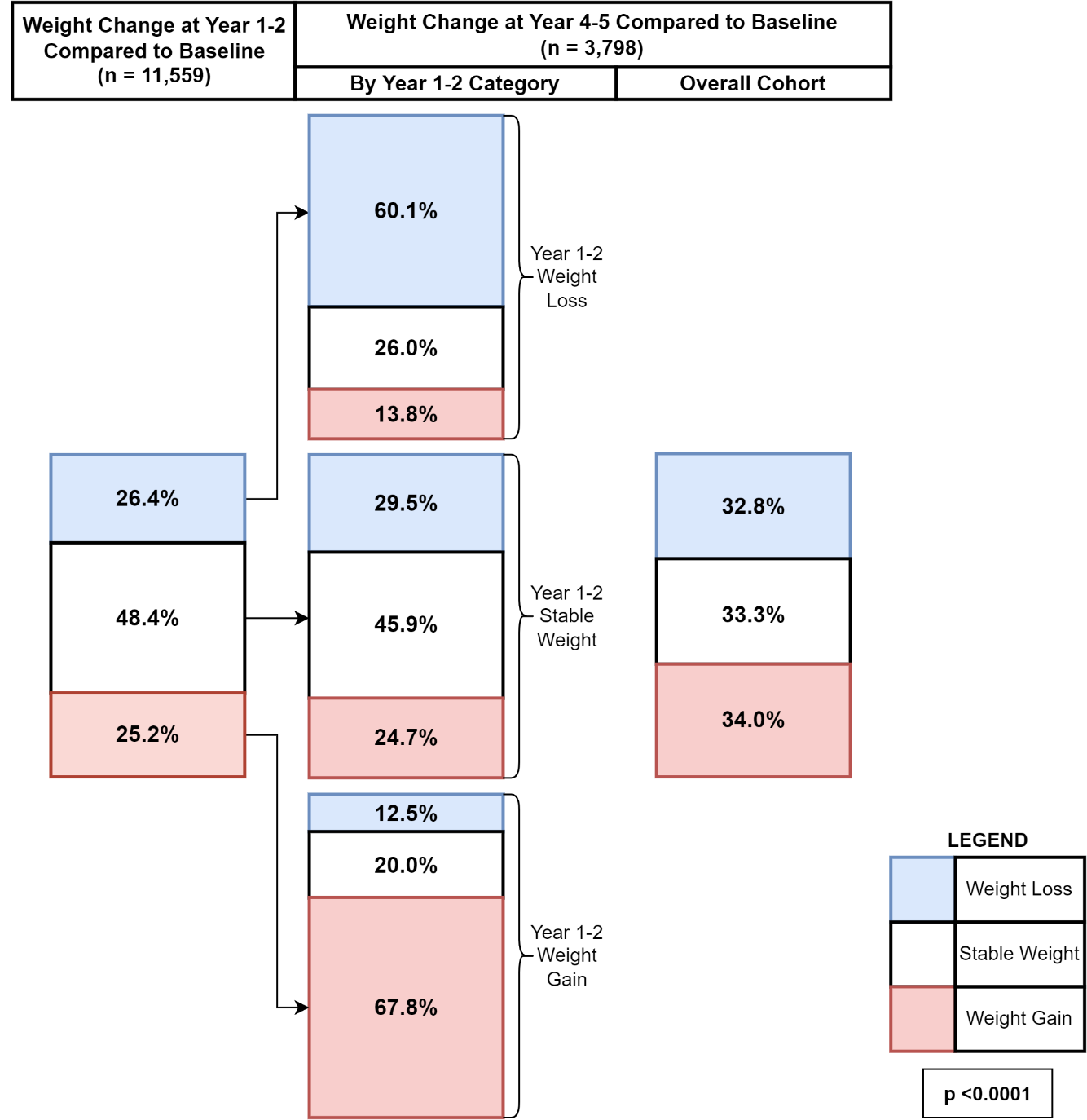


Weight categories are defined as weight loss (≥5% weight decrease; blue), stable weight (<5% weight decrease or increase; white), and weight gain (≥5% weight increase; red). Values in the boxes show percentage of patients in each category. The leftmost column shows the overall distribution of weight category at year 1-2. The middle column shows the distribution of weight category at year 4-5, stratified by weight category at year 1-2. The rightmost column shows the overall distribution of weight category at year 4-5. P value is by a chi-square test comparing distribution of weight category in year 4-5, stratified by weight category at year 1-2.

**Supplemental Figure 5. Weight trends at year 1-2 and year 4-5 follow-up compared to baseline in patients with nonalcoholic fatty liver disease - sensitivity analysis requiring 10.0% body mass index change**


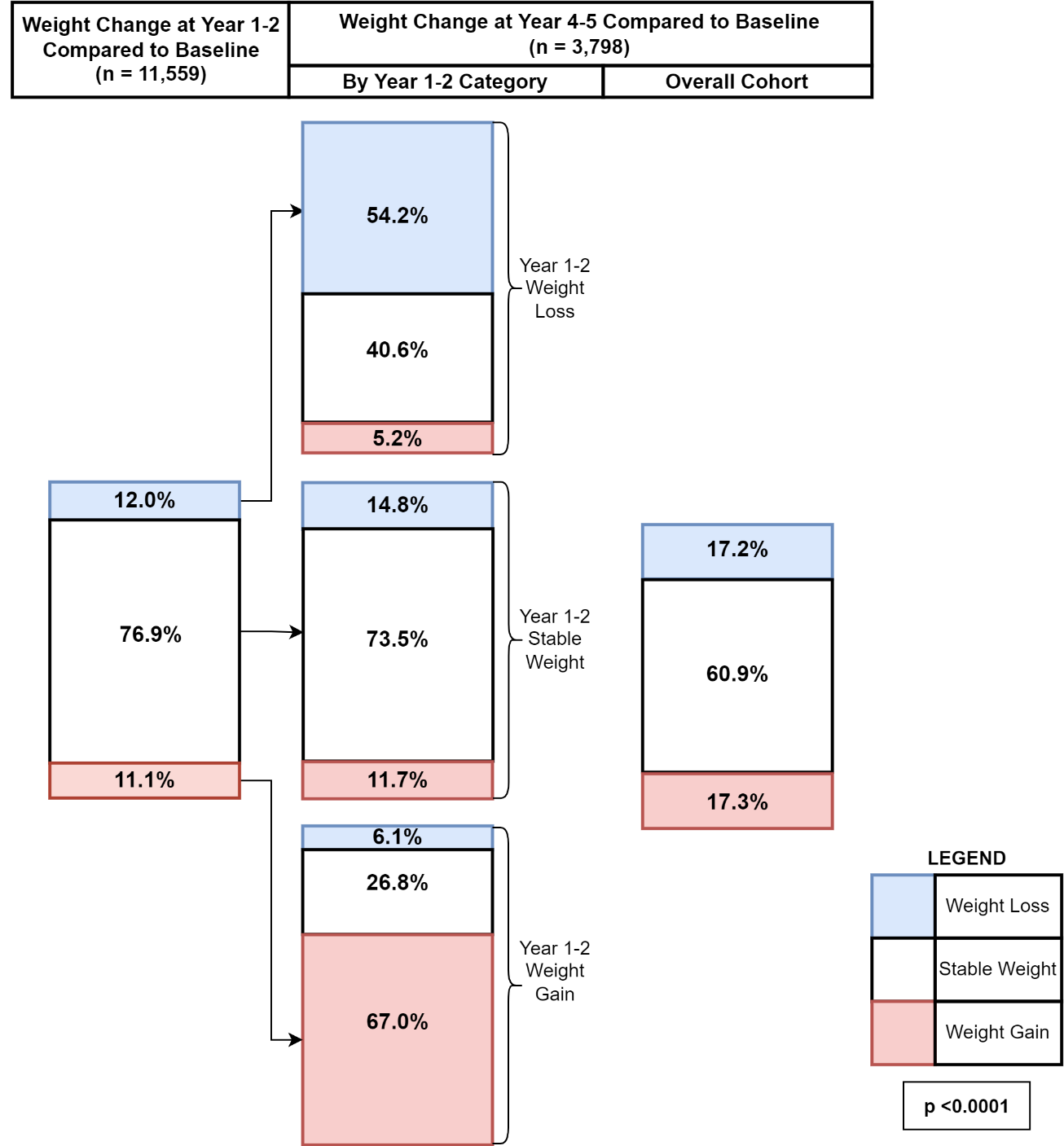


Weight categories are defined as weight loss (≥10% weight decrease; blue), stable weight (<10% weight decrease or increase; white), and weight gain (≥10% weight increase; red). Values in the boxes show percentage of patients in each category. The leftmost column shows the overall distribution of weight category at year 1-2. The middle column shows the distribution of weight category at year 4-5, stratified by weight category at year 1-2. The rightmost column shows the overall distribution of weight category at year 4-5. P value is by a chi-square test comparing distribution of weight category in year 4-5, stratified by weight category at year 1-2.

**Supplemental Figure 6. Weight trends at year 1-2 and year 4-5 follow-up compared to baseline in patients with nonalcoholic fatty liver disease - sensitivity analysis excluding patients who received medications that can cause weight changes during the follow-up period**


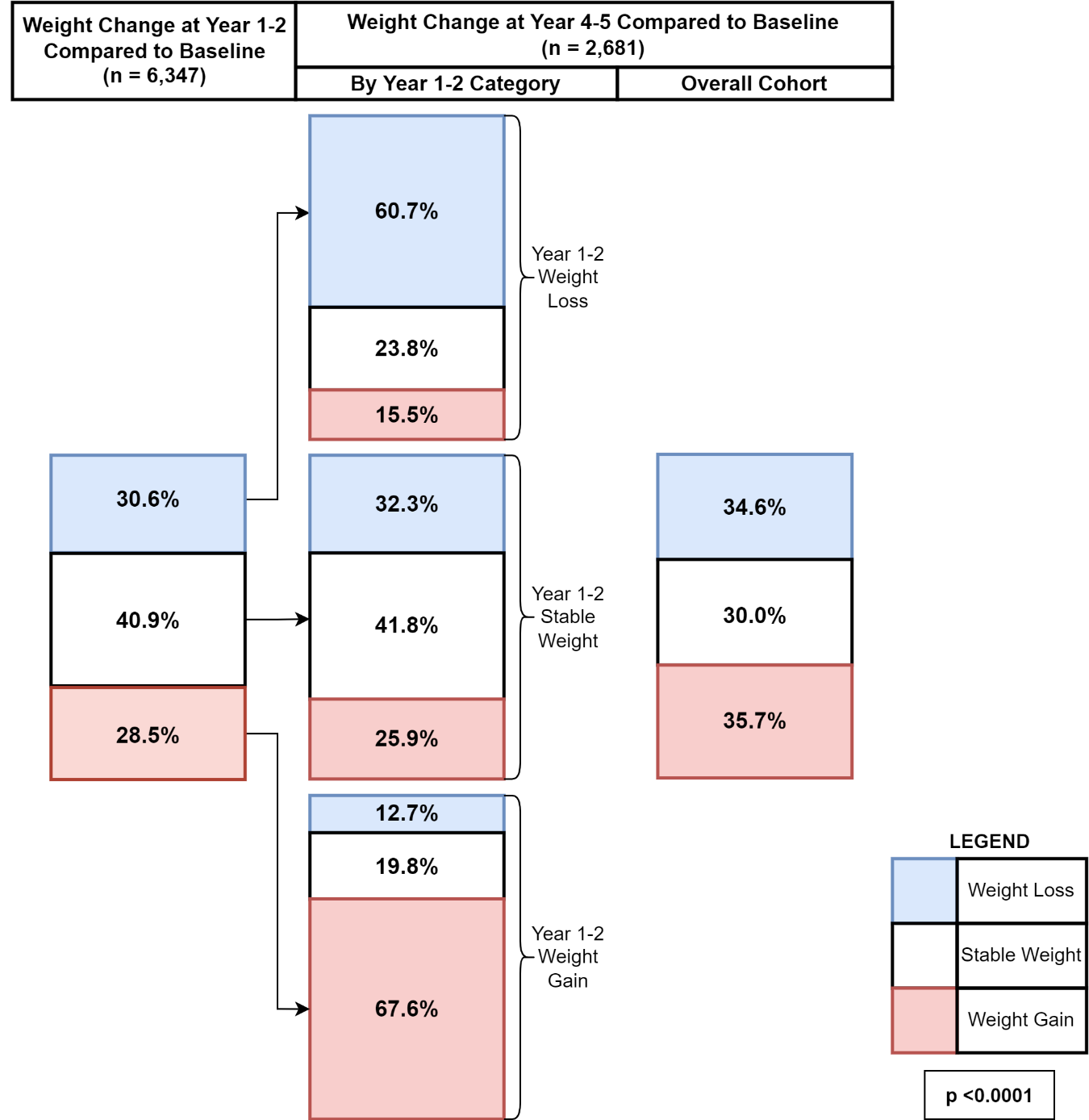


Weight categories are defined as weight loss (≥5% weight decrease; blue), stable weight (<5% weight decrease or increase; white), and weight gain (≥5% weight increase; red). Values in the boxes show percentage of patients in each category. The leftmost column shows the overall distribution of weight category at year 1-2. The middle column shows the distribution of weight category at year 4-5, stratified by weight category at year 1-2. The rightmost column shows the overall distribution of weight category at year 4-5. P value is by a chi-square test comparing distribution of weight category in year 4-5, stratified by weight category at year 1-2.
